# A deep learning model for diagnosing dystrophinopathies on thigh muscle MRI images

**DOI:** 10.1186/s12883-020-02036-0

**Published:** 2021-01-11

**Authors:** Mei Yang, Yiming Zheng, Zhiying Xie, Zhaoxia Wang, Jiangxi Xiao, Jue Zhang, Yun Yuan

**Affiliations:** 1 Department of Neurology, Peking University First Hospital, Beijing, China; 2 Academy for Advanced Interdisciplinary Studies, Peking University, Beijing, China; 3 Department of Radiology, Peking University First Hospital, Beijing, China

**Keywords:** Magnetic Resonance Imaging, Muscular Diseases, Deep Learning, Computer-Assisted Diagnosis

## Abstract

**Background:**

Dystrophinopathies are the most common type of inherited muscular diseases. Muscle biopsy and genetic tests are effective to diagnose the disease but cost much more than primary hospitals can reach. The more available muscle MRI is promising but its diagnostic results highly depends on doctors’ experiences. This study intends to explore a way of deploying a deep learning model for muscle MRI images to diagnose dystrophinopathies.

**Methods:**

This study collected 2536 T1WI images from 432 cases who had been diagnosed by genetic analysis and/or muscle biopsy, including 148 cases with dystrophinopathies and 284 cases with other diseases. The data was randomly divided into three sets: the data from 233 cases were used to train the CNN model, the data from 97 cases for the validation experiments, and the data from 102 cases for the test experiments. We also validated our models expertise at diagnosing by comparing the model’s results on the 102 cases with those of three skilled radiologists.

**Results:**

The proposed model achieved 91% (95% CI: 0.88, 0.93) accuracy on the test set, higher than the best accuracy of 84% in radiologists. It also performed better than the skilled radiologists in sensitivity : sensitivities of the models and the doctors were 0.89 (95% CI: 0.85 0.93) versus 0.79 (95% CI:0.73, 0.84; *p* = 0.190).

**Conclusions:**

The deep model achieved excellent accuracy and sensitivity in identifying cases with dystrophinopathies. The comparable performance of the model and skilled radiologists demonstrates the potential application of the model in diagnosing dystrophinopathies through MRI images.

## Background

Dystrophinopathies are the most common muscular dystrophies caused by mutation of the dystrophin gene. Severe Duchenne muscular dystrophy (DMD) and milder Becker muscular dystrophy(BMD) are the two typical dystrophinopathies. These diseases are characterized by progressive muscle weakness and muscular atrophy, mostly leading to cardiopulmonary failure and even death. Glucocorticoid therapy has been proved to be able to stabilize or improve muscle strength. When ambulation becomes more marginal, the benefit might be more limited [1], which implies timely and accurate diagnosis is crucial.

Two commonly used diagnostic approaches for dystrophinopathies are genetic testing and muscle biopsy. Although both techniques have exhibited specific expertise, they still need to be tailored to dystrophinopathies. Multiplex ligation-dependent probe amplification (MLPA), a fast and convenient gene testing method, has gained global popularity, but can only detect 70% cases of dystrophinopathies [2, 3] due to different mutation types in the dystrophin gene. The next-generation sequencing can identify about 95% cases with dystrophinopathies [3], but its complex operation and prohibitive price hinder its spread in less-developed countries and regions. Immunohistochemical staining, a muscle biopsy method, can achieve 100% sensitivity for identifying DMD while only 73% for BMD [4], and its invasive and complex technique may prevent ordinary hospitals from implementing it.

Magnetic resonance imaging (MRI) has attracted physicians as a promising alternative to diagnose dystrophinopathies, especially those who work with limited health recourses, thanks to its noninvasive and convenient operation. Several studies have described MRI findings in muscles of dystrophinopathies that indicated a distinct pattern of fatty infiltration [5–9]. This pattern can be used to assess disease progression and guide following diagnosis [10]. Dam et al. evaluated muscle MRI/CT to assess BMDs, and obtained a sensitivity of 91% [11]. Zheng et al. proposed the trefoil with single fruit sign as an imaging marker for diagnosing dystrophinopathies and achieved a specificity of 99.2% [12].

These fruitful studies have propelled researchers to further improve the accuracy and reliability of MRI imaging, but a fundamental difficulty stands in the way. Current assessment of dystrophinopathies through MRI images is to identify fat infiltration in muscles by doctors. It highly depends on personal experiences and can easily lead to misdiagnosis, given that similar patterns that some other muscular dystrophies exhibit might perplex junior physicians. Therefore, an more objective analysis method is required. Deep learning seems to be a perfect choice. It extracts informative features from medical data to classifies certain patterns, thus to present effective and objective diagnosis. Deep learning methods have been serving in clinical practice as a valuable assistance [13, 14]. For example, convolutional neural networks (CNNs) have gained appealing results on several medical MRI datasets [15–17]. However, most published works on muscle MRI images focused on segmentation of different muscle regions [18–20], and barely noticed patterns of diseases. Given CNNs’ classifying power, their ability of identifying dystrophinopathies in muscle MRI images need to be explored.

This study incorporates a CNN network to MRI imaging assessment, and intends to test and verify the reliability of diagnosis on dystrophinopathies through muscle MRI. A comparison experiment is conducted to demonstrate the diagnostic power of the proposed CNN-based approach versus radiologists.

## Methods 

**Fig. 1:**
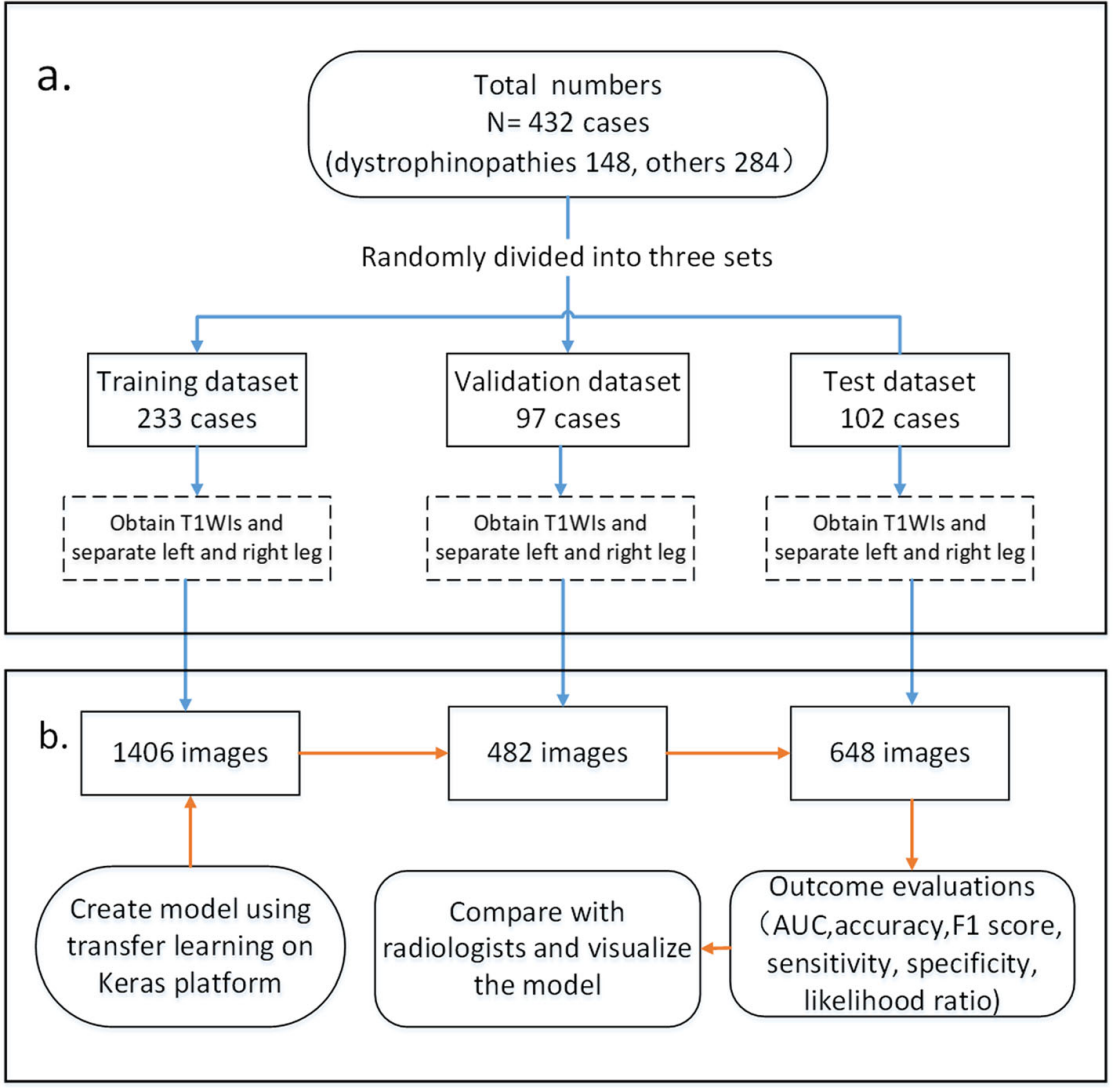
Workflow Diagram. **a** Flowchart of obtaining the images of three data sets. **b** Flowchart of constructing the CNN model using the three datasets

The workflow of the proposed approach is depicted in Fig. 1.

### Study population

We collected MRI data from 432 cases including 148 cases with dystrophinopathies and 284 cases with other muscle diseases (other types of muscular dystrophy, myositis and neurogenic diseases) in the control group. The 432 patients were all ambulatory during the MR examinations.

The 148 cases with dystrophinopathies were all male, with an average age of 14.8 ± 6.6 years (8–57 years), and an average course of 9.4 ± 6.7 years (2.0-51.5 years). The average course of 117 DMD patients was 7.3 ± 2.5 years (2.0-12.8 years), and the average course of 31 BMD patients was 17.1 ± 10.6 years (3.9–51.5 years). The 284 cases in the control group consisted of e 164 males and 120 females (male to female ratio 1.4:1), with an average age of 36.8 ± 16.9 years (9–81 years) and the average course was 5.8 ± 5.9 years (1month-22 years).

We randomly divided the cases into three sets: training, validation, test. The resulted training set included 233 cases, the validating 97 and the testing 102. Each case corresponded to multi-T1WIs (T1-weighted images) of cross-sectional MRI scanning proximally at the level of the mid-thigh. Muscle MRI examinations at 1.5 or 3.0 T (GE Healthcare, Waukesha, WI, USA) were performed with the following sequences: axial T1-weighted spin echo series with 450/12 (repetition time, ms/echo time, ms). All cases had been diagnosed by electromyography, the gene sequencing, test for myositis associated or specific autoantibodies, or muscle biopsy.

### ROI localization

We acquired the region of interest (ROI) in each original MRI image using the Otsu threshold and the adaptive window method. First, the largest connected parts in the image indicating the hip area was identified, and then two regions of the left and the right legs connected by the hip could be separated by the Otsu threshold selection [21]. Second, an adaptive window was determined to search a minimal rectangle that contains the ROI in each image while eliminating invalid background area as much as possible.

### CNN Model

We compared the performance of classical CNN networks like Inception-V3 [22], Resnet50 [23], VGG-19 [24] and DenseNet [25] in the dataset. As shown in Table 1, Resnet50 achieved excellent performance in several metrics, so we adopted ResNet50 as our base model. The ResNet50 network introduces a residual module to solve the problems such as gradient degradation and training difficulty caused by the increasing depth of CNNs.

**Table 1:** Image classification comparison: classification results are reflected by accuracy, specificity and sensitivity

**Model**	**Accuracy**	**Specificity**	**Sensitivity**	**AUC**
**VGG-19** [24]	0.87 (95%CI: 0.84, 0.89)	**0.98 **(95%CI: 0.96, 0.99)	0.66 (95%CI: 0.59, 0.72)	0.91
**ResNet50** [23]	**0.91 **(95%CI: 0.88, 0.93)	0.92 (95%CI: 0.89, 0.94)	**0.89 **(95%CI: 0.85, 0.93)	**0.98**
**DenseNet201** [25]	0.90 (95%CI: 0.87, 0.92)	**0.98 **(95%CI: 0.96, 0.99)	0.74 (95%CI: 0.68, 0.79)	0.96
**DenseNet121** [25]	0.88 (95%CI: 0.85, 0.91)	0.94 (95%CI: 0.91, 0.96)	0.78 (95%CI: 0.72, 0.83)	0.94
**Inception-V3** [22]	0.90 (95%CI: 0.87, 0.92)	0.94 (95%CI: 0.91, 0.96)	0.83 (95%CI: 0.77, 0.87)	0.96

**Fig. 2:**
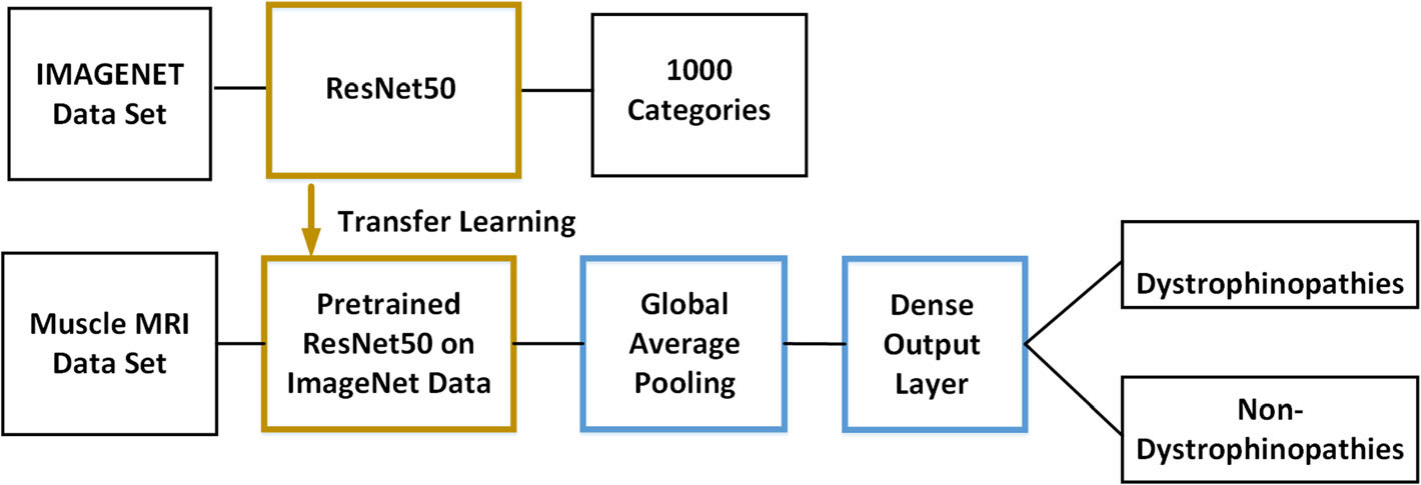
Schematic of a convolutional neural networks with transfer learning. First, the ResNet50 model is trained on the ImageNet dataset of 1000 categories; Second, the convolutional layers are frozen and transferred into a new network; Third, the fully connected layers are retrained through the input of dystrophinopathies T1W1 images; Finally, the model outputs binary classification results

Given limited labeled clinical images that we had, we had to circumvent traditional training procedures that usually require ten thousands to millions of labeled images while preventing the training from over-fitting. Therefore, we retrofitted the ResNet50 architecture and pre-trained it on the ImageNet dataset [26]. As shown in Fig. 2, the convolutional layers trained on ImageNet are frozen and used as fixed feature extractors; the global average pooling layer and the fully connected layers are recreated and retrained to form a new model that can classify our specific medical categories. Also, binary-entropy was chosen as the loss function, and stochastic gradient descent [27] for optimization. The training was performed with a learning rate of 1e-4 and a batch size of 32 until it converged. The networks were established on Keras deep learning library with Tensorflow backend.

### Evaluation metrics

To validate the proposed model, we compared its results with those from three radiologists specialized at muscle diseases: a resident with five years’ experience, an attending doctor with six years’ experience, and a resident with three years of experience. They were all provided only with T1WI images without access to any information about the cases.

Fleiss Kappa [28] was adopted to measure the inter-examiner agreement. In this study, the indication of different ranges of Kappa values were determined as: <0, no agreement; 0–0.20, slight agreement; 0.21–0.40, fair agreement; 0.41–0.60; moderate agreement; 0.61–0.80 substantial agreement; and 0.81–1.00, nearly perfect agreement.

We also used the notions of sensitivity, specificity and accuracy to evaluate the diagnostic accuracy of the proposed model. Sensitivity means the percent of true positives that are correctly identified. Specificity refers to the percent of true negatives that are correctly identified. Accuracy of a classifier represents the overall percentage of correct classifications.

F1 score was also used to comprehensively compare the results between the model and the doctors. F1 score is the harmonic mean of Precision and Recall, including positive likelihood ratio [29] and negative likelihood ratio. The positive likelihood ratio describes how well a test performs in identifying a disease state. It is obtained by the probability of a positive test result in the presence of the disease divided by the probability of a positive test result in the absence of the disease. The higher the positive likelihood ratio, the better the model performs in identifying disease. The negative likelihood ratio indicates how well a model excludes a disease state. The lower it is, the better the model performs on excluding a disease.

### Decision‐making basis

Machine learning systems are often regarded as black boxes given their mysterious decision-making processes that people cannot inspect. To present a more comprehensible process, we applied class activation mapping (CAM) to visualize the decision basis of the network [30]. The features of the last convolutional layer in the CNNs were linearly weighted to highlight the regions that could determine the model’s different classification results.

## Results

### Patient and image characteristics

We finally obtained 2536 images from 432 cases that held sufficient quality and met diagnostic standards, and some images were deleted where the distances between the two legs were too short for separation. Among them, 1406 from 233 cases were used for training, 482 from 97 cases for validation, and 648 from 102 cases for test the performance of the model. The detailed information of these images is listed in Table 2.

**Table 2:** The detailed information about patients and image of various diseases in different data sets

***Cases /Image size***	***Training***	***Validation***	***Test***	***Total***
***Muscular disease***				
***DMD***	71/480	26/120	20/130	**117/730**
***BMD***	9/76	5/40	17/98	**31/214**
***LGMD2A***	20/86	8/32	2/8	**30/126**
***LGMD2B***	12/38	8/32	2/8	**22/78**
***LGMD2C***	2/8	0	0	**2/8**
***LGMD2D***	15/56	6/24	3/12	**24/92**
***LGMD2E***	8/32	3/12	4/16	**15/60**
***LGMD2I***	10/64	5/20	2/12	**17/96**
***Bethlem myopathy***	4/16	1/4	1/4	**6/24**
***FSHMD***	8/32	2/8	2/8	**12/48**
***Congenital myopathies***	8/32	2/8	2/8	**12/48**
***GNE myopathy***	5/40	3/18	0	**8/58**
***MADD***	15/58	6/24	5/20	**26/102**
***Myositis***	18/144	15/92	24/196	**57/432**
***Neurogenic***	28/244	7/48	18/128	**53/420**
***Total***	**233/1406**	**97/482**	**102/648**	**432/2536**

*Abbreviations*: *DMD *Duchene muscular dystrophy, *BMD *Becker muscular dystrophy, *LGMD *limb-girdle muscular dystrophy, *FSHMD *facioscapulohumeral muscular dystrophy, *MADD *multiple acyl coenzyme A dehydrogenation deficiency

### Comparison of the model with human experts

**Fig. 3:**
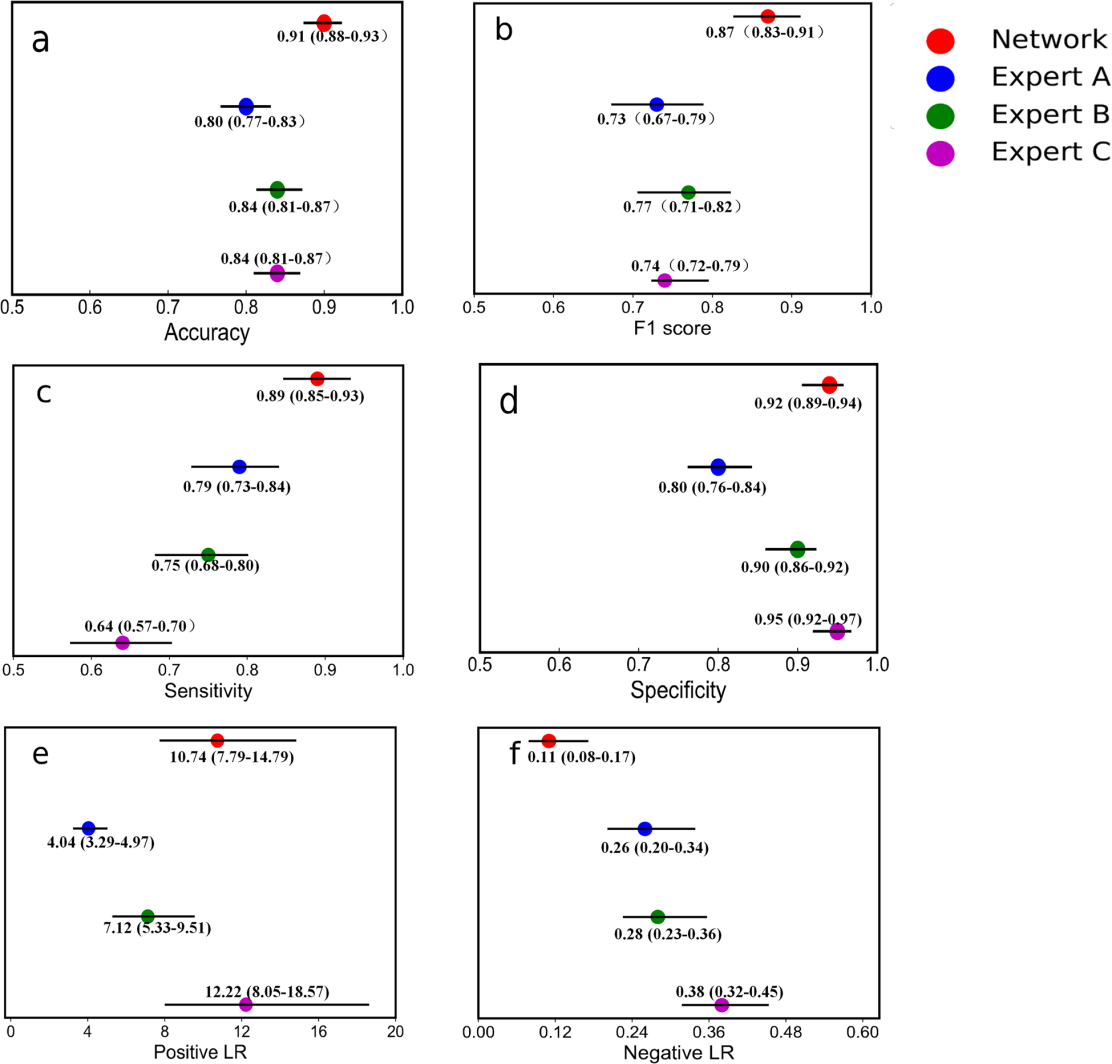
Various metrics of experts and networks for dystrophinopathies diagnosis.** a**, Accuracy. **b**, F1 score. **c**, Sensitivity. **d**, Specificity. **e**, Positive LR. **f**, Negative LR. The legend of each subplot reports the detailed numerical results

The comparison results between the model and the physicians are depicted in Fig. 3.

**Fig. 4:**
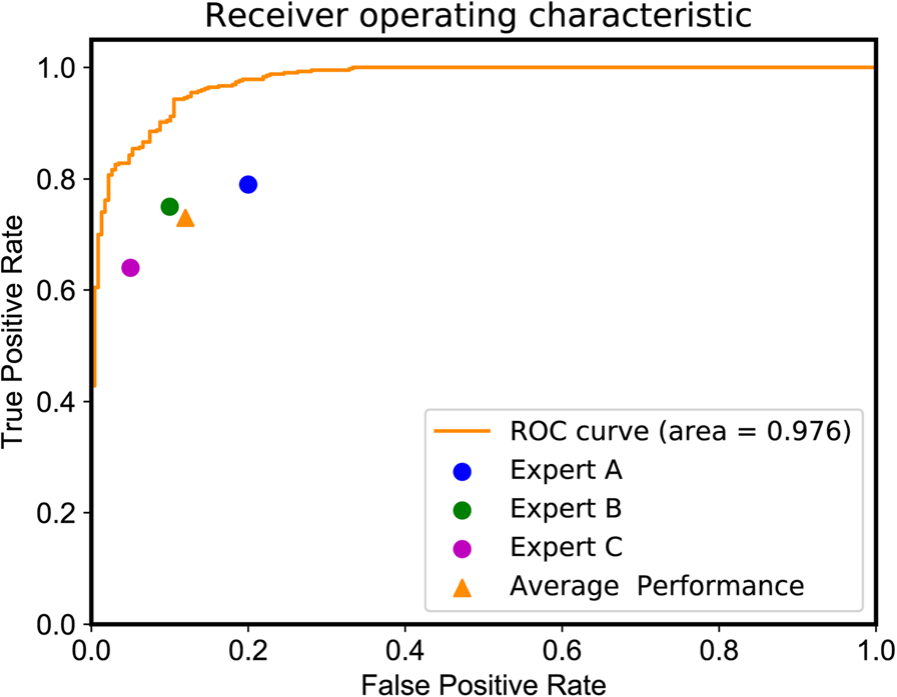
Receiver operating characteristic curve obtained using the convolution network. The receiver operator characteristic (ROC) area under the curve (AUC) is 0.98, and the orange triangle refers to the average performance of the experts. Circles and the triangle are all below the curve

The accuracies of the three physicians were 0.80 (95% CI 0.77–0.83), 0.84 (95% CI 0.81–0.87), and 0.84 (95% CI 0.81–0.87), and the inter-examiner agreement of them was substantial (Kappa 0.628). The accuracy of the model was 0.91 (95% CI 0.88–0.93), which was higher than those of the doctors. Other metrics of the model, including F1 score, sensitivity, specificity, and likelihood ratio, were also better than those of the radiologists. Figure 4 displays the sensitivities and specificities of the experts on the ROC curve of the trained model. The area under the ROC curve was 0.98, and the performance was comparable between the model and the doctors.

### Visualization of the model

**Fig. 5:**
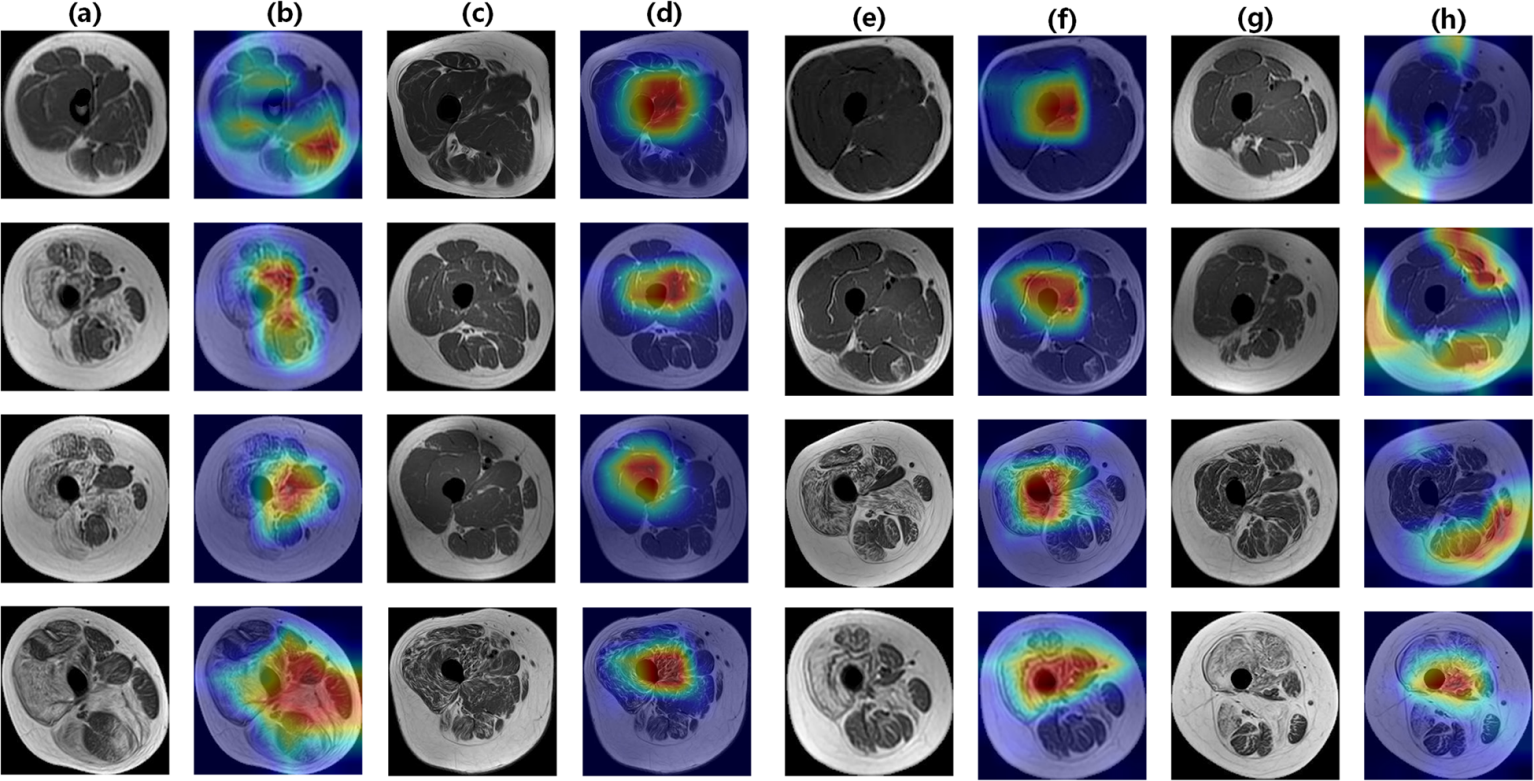
Saliency maps of the correctly diagnosed/the misdiagnosed dystrophinopathy/non- dystrophinopathy samples. Colors ranging from red to blue indicates the importance of image regions from high to low. **a** original images of samples correctly diagnosed as dystrophinopathy by the CNNs; **c** original images of correctly diagnosed as non-dystrophinopathy samples; **e** original images of incorrectly diagnosed as dystrophinopathy samples; **g** original images of incorrectly diagnosed as non-dystrophinopathy samples (**b**, **d**, **f**, **h**) are the corresponding saliency maps

The visualization of the model’s decision basis is shown in Fig. 5. The model paid different attentions to different regions of the input images. Regions in red and yellow were emphasized and could influence the results. In the cases with dystrophinopathies, the model tended to focus on multiple localized regions, whereas in cases diagnosed with non- dystrophinopathies, the model’s region of interest was concentrated near the bones.

### Classification on DMD and BMD

Since dystrophinopathies have two clinical subtypes, DMD and BMD, and the corresponding clinical treatments of them are different, we further investigated the sensitivity of the model to DMD and BMD.

The test set included a total of 130 images from patients with DMD, 98 images from patients with BMD, and 420 images from patients with other diseases. As shown in Table 3, the 123 images from the DMD group were classified as dystrophinopathies, and the 81 images from the BMD group were classified as dystrophinopathies. The sensitivity of this model to DMD (94%) was higher than that to BMD (82%).

**Table 3:** Classification results about DMD and BMD

Predicted	Dystrophinopathy	Non-dystrophinopathy
Actual		
**DMD**	123	7
**Non-dystrophinopathy**	35	385
**BMD**	81	17
**Non-dystrophinopathy**	35	385

## Discussion

The presented study explored the possibility of deep-learning methods of detecting dystrophinopathies on MRI images. The comparison experiments demonstrated that the proposed model can identify dystrophinopathies on MRI images with an AUC of 0.98, accuracy of 91%, and achieve similar specificity and higher sensitivity compared with skilled radiologists. Our research expands MRI’s potential in detecting dystrophinopathies, thus can further spread MRI as a low-cost diagnosis method to less-developed regions.

More than the excellent performances that other deep learning methods have achieved [31, 32], our model exploits extra values of machine-learning methods for clinical practice. First, our model provides an effective way of training a deep-learning model with limited MRI images. Collecting enough data of dystrophinopathies is crucial to doctors as well as to traditional deep-learning models, because doctors need to summarize rules and characteristics through years of practice on large amount of data, just like a normal deep-learning model dose. Our approach integrates transfer learning strategy, a proved highly effective technique for limited data [33, 34], to train a model in a short time with limited samples. Plus, the performance of our model would be enhanced with continuous accumulation of clinical data. Second, our model provides an objective way of diagnosing dystrophinopathies. Diagnosing dystrophinopathies through MRI images is to assess the degree of muscle fat infiltration. Given no quantitative standard for the degrees, different doctors would present different diagnoses. Luckily, the deep-learning model can tackle this problem, because features it extracts from MRI images are independent from radiologists’ judgments, leading to a more objective and less bias-prone results. Third, our model also provides extra ability of interpreting decision-making process. This visualization suggested that the network is not focused on specific regions like experts, and it has its own way to distinguish between dystrophinopathies and non-dystrophinopathies, a pattern that does not exactly match the experience of clinical experts.

To the best of our knowledge, our study incorporates the largest number of MRI images of cases with dystrophinopathies for a deep learning model. All cases had been diagnosed by muscle biopsy and genetic examination. The categorizing followed the popular principle: the validation and the test sets hold similar sizes that are smaller than that of the training set [35–37]. The study also adhered to the premise that MRI images from one case cannot exist in different sets. To exploit the model’s feasibility, we included more diseases in the control group, like the limb-girdle muscular dystrophy with similar MRI characteristics, the inflammatory myopathy, and other muscular diseases. In addition, our model has a higher sensitivity in diagnosing DMD compared with BMD, which may be related to the fact that BMD patients correspond to a mild lesion degree of MRI imaging.

Several studies have implied an increasing awareness of the value of MRI in muscular dystrophy. Carlier et al. indicated that MRI can be utilized in the diagnostic workup, and believed that the potential of MRI to diagnose muscular dystrophy should be maximized [38]. Kim et al. reviewed the development of MRI technology, and analyzed its effect on MRI’s application in muscular dystrophies [39]. They concluded that further investigation of techniques might provide new opportunities for convincing diagnosis. Cai et al. proposed a deep-learning method and achieved an accuracy of 91.7% for classifying muscle diseases in 42 cases. But the data used in their work were acquired through chemical shift-based water-fat separation MR imaging [40]. Although the sequence can present clearer results of fat infiltration in muscles, it requires higher technique of imaging, which obviously restricts its clinical application. In contrast, the T1WIs are more easily available in clinical practice while well revealing the fat infiltration in muscles. They have been widely used in clinical trials [41–43]. Our study is the first to demonstrate that T1WI images can be used by the deep learning model, and the improved sensitivity echoes the aim of preliminary screening dystrophinopathies using MRI.

Some limitations in our study need to be considered. First, our model only outputs binary results of classification, and diversified diagnosis, like identifying muscle dystrophies or inflammatory myopathies by one approach, needs to be further addressed. Second, reasonable inclusion of the fat-saturated T2-weighted sequence information might improve the diagnosis results and sensitivity of the model. Third,our study only deals MRI images of thigh areas, without 3D images of the whole leg or other parts that could contain lesions, like the shank or the upper limb. Future work should include more parts of possible lesions to improve the model’s generalizability. Fourth the research data were collected from a single source. Since MRI imaging parameters from different sources usually varies, and data from more sources should be incorporated. Last, our work did not include a control sample of healthy individuals. In fact, healthy patients rarely initiate muscle MRI, so we could not collect adequate healthy control data for study. Besides, it is simpler to distinguish healthy people from patients in the clinic. But we will include a sufficient number of heathy human MRI images for comparative study in our follow-up work.

## Conclusions

In conclusion, our results suggest that the deep learning model presents comparable performance compared with experience radiologists. Our study indicates a promising future of deep-learning methods of diagnosing muscle diseases, especially dystrophinopathies, through MRI images,. With the development of deep learning methods, the potential of MRI in detecting dystrophinopathies can be expanded, thus we can further spread MRI to less-developed regions as a low-cost diagnosis method.

## Data Availability

The datasets used and analyzed during the current study available from the corresponding author on reasonable request.

## Abbreviations

DMD: Duchene muscular dystrophy
BMD: Becker muscular dystrophy
MLPA: Multiplex ligation-dependent probe amplification
ROC: Receiver operator characteristic
MRI: Magnetic resonance imaging
T1WI: T1-weighted images
CNN: Convolutional neural network
